# Patient and provider perceptions of a peer-delivered intervention (‘*Khanya*’) to improve anti-retroviral adherence and substance use in South Africa: a mixed methods analysis

**DOI:** 10.1017/gmh.2022.47

**Published:** 2022-08-26

**Authors:** Alexandra L. Rose, Jennifer M. Belus, Abigail C. Hines, Issmatu Barrie, Kristen S. Regenauer, Lena S. Andersen, John A. Joska, Nonceba Ciya, Sibabalwe Ndamase, Bronwyn Myers, Steven A. Safren, Jessica F. Magidson

**Affiliations:** 1 Department of Psychology, University of Maryland, College Park, MD, USA; 2 Department of Medicine, Swiss Tropical and Public Health Institute, Basel, Switzerland; 3 Westat, Rockville, MD, USA; 4 Global Health Section, Department of Public Health, University of Copenhagen, Copenhagen, Denmark; 5 HIV Mental Health Research Unit, University of Cape Town, Cape Town, South Africa; 6 South African Medical Research Council, Alcohol, Tobacco, and Other Drug Research Unit, Cape Town, South Africa; 7 Curtin enAble Institute, Faculty of Health Sciences, Curtin University, Bentley, Australia; 8 Division of Addiction Psychiatry, Department of Psychiatry and Mental Health, University of Cape Town, Cape Town, South Africa; 9 Department of Psychology, University of Miami, Coral Gables, FL, USA

**Keywords:** HIV, mindfulness, peer, South Africa, substance-related disorders

## Abstract

**Background:**

Despite a high prevalence of problematic substance use among people living with HIV in South Africa, there remains limited access to substance use services within the HIV care system. To address this gap, our team previously developed and adapted a six-session, peer-delivered problem-solving and behavioral activation-based intervention (*Khanya*) to improve HIV medication adherence and reduce substance use in Cape Town. This study evaluated patient and provider perspectives on the intervention to inform implementation and future adaptation.

**Methods:**

Following intervention completion, we conducted semi-structured individual interviews with patients (*n* = 23) and providers (*n* = 9) to understand perspectives on the feasibility, acceptability, and appropriateness of *Khanya* and its implementation by a peer. Patients also quantitatively ranked the usefulness of individual intervention components (problem solving for medication adherence ‘Life-Steps’, behavioral activation, mindfulness training, and relapse prevention) at post-treatment and six months follow-up, which we triangulated with qualitative feedback to examine convergence and divergence across methods.

**Results:**

Patients and providers reported high overall acceptability, feasibility, and appropriateness of *Khanya*, although there were several feasibility challenges. Mindfulness and Life-Steps were identified as particularly acceptable, feasible, and appropriate components by patients across methods, whereas relapse prevention strategies were less salient. Behavioral activation results were less consistent across methods.

**Conclusions:**

Findings underscore the importance of examining patients’ perspectives on specific intervention components within intervention packages. While mindfulness training and peer delivery models were positively perceived by consumers, they are rarely used within task-shared behavioral interventions in low- and middle-income countries.

## Introduction

Globally, access to substance use (SU) interventions remains limited, largely due to a lack of trained mental health providers (Bruckner *et al*., 2011). The gap between the behavioral health care need and available services has led to the development of innovative delivery models, including task sharing, or the distribution of behavioral health care tasks to non-specialist workers (Hoeft *et al*., 2018; Singla *et al*., 2018). The degree to which users of these interventions view them as *acceptable* (satisfactory in terms of content and complexity), *feasible (*practical to deliver), and *appropriate* (useful for their setting), is key to service users’ uptake and engagement in the intervention and providers’ continued implementation of the innovation (Proctor *et al*., 2011, 2013; Mira *et al*., 2019). Yet, there remains a relative lack of evidence on patient and provider perspectives on the implementation outcomes of SU interventions delivered via task-sharing in low- and middle-income countries (LMICs). This represents a barrier to further intervention adaptation for scale up of services.

Further understanding patient and provider perspectives on the implementation outcomes of existing interventions may help inform future adaptations and intervention development. Within countries like South Africa, it is particularly important to understand perspectives on intervention components that are helpful for people living with HIV (PLWH). South Africa has the largest number of PLWH globally, more than 7.5 million (Kim *et al*., 2021). There is also a high prevalence of SU problems among PLWH in the country (Kader *et al*., 2014; Necho *et al*., 2020), which has led to the development of interventions to target both psychological and health outcomes within this population (Kekwaletswe and Morojele, 2014; Myers *et al*., 2018; Magidson *et al*., 2020*b*).

Prior research has documented far more intervention packages than intervention components, meaning many intervention packages actually utilize similar components in various configurations (Domhardt *et al*., 2019; Boustani *et al*., 2020). Therefore, there may be a greater value from examining the implementation outcomes of individual components than of the overall packages. Examining the implementation outcomes of specific components may lead to actionable findings not only relevant to that intervention package but also to others that include the same component. Examining how specific components are perceived may also help in developing more streamlined or personalized interventions. Moreover, when examining patient and provider perspectives on interventions, qualitative and quantitative data may add different information and be best used in combination (O'Cathain *et al*., 2010).

In addition to adequately understanding perceptions of interventions and their components, it is important to understand perspectives on delivery models. Peer delivery models, in which an individual with lived experience of a condition delivers the intervention, are becoming increasingly common in high-income countries, particularly for SU interventions (Eaton *et al*., 2019; Shalaby and Agyapong, 2020). Yet few studies in LMICs have used peers to deliver task-shared interventions. Consequently, there is a knowledge gap on patient and provider perspectives of the acceptability, feasibility, and appropriateness of peer-delivered SU interventions in these settings (Satinsky *et al*., 2021).

In a pilot randomized type 1 hybrid effectiveness-implementation trial of *Khanya*, an integrated, peer-delivered, behavioral activation (BA) and problem solving-based intervention to address SU and HIV medication adherence, participants experienced significant improvements in ART adherence compared to enhanced treatment as usual (Magidson *et al*., 2021). Participants also reported very high levels of acceptability, feasibility, and appropriateness of *Khanya* on a quantitative assessment (*M* = 2.98/3 for both feasibility and acceptability; 2.94/3 for appropriateness (Magidson *et al*., 2021). The current study presents the primary qualitative aim of this trial (Magidson *et al*., 2021) in which we examine patient and provider perspectives on the feasibility, acceptability, and appropriateness of *Khanya* as an overall intervention and its delivery by a peer. Using mixed methods to triangulate qualitative and quantitative data, we also examine patient perspectives on specific intervention components (i.e. Life-Steps, BA, mindfulness training, relapse prevention strategies). Findings may aid future adaptations of *Khanya* and/or interventions using similar components.

## Methods

### Participants and procedures

As reported on previously (Magidson *et al*., 2020*b*, 2021), a pilot randomized hybrid type 1 effectiveness-implementation trial (*N* = 61) was conducted to evaluate the *Khanya* intervention package; between August 2018 and October 2019, 30 participants were randomized to receive *Khanya*, and 31 participants to receive enhanced treatment as usual (facilitated referral to a local SU treatment program). Participants were recruited from a large, public primary care clinic in Cape Town. Participants were eligible for the trial if they were: (1) on antiretroviral treatment (ART) for HIV; (2) 18 to 65 years old; (3) reported at least moderate SU; (4) and had ART non-adherence in the past three months. For additional detail on *Khanya's* development and clinical outcomes, please see prior publications (Magidson *et al*., 2019, 2020*a*, 2020*b*, 2020*c*, 2021). Data used in the current study aim were collected at visits conducted at three- (post-treatment) and six- (follow-up) months after baseline. All procedures were approved by the University of Cape Town Human Research Ethics Committee (HREC 187/2018) using an IRB Authorization Agreement (IAA) with the University of Maryland College Park.

### *Khanya* intervention

*Khanya* was delivered by a single local peer interventionist with shared lived experience of SU and prior training in BA, over six sessions in a three-month period. *Khanya* included intervention components selected on two main criteria: (1) prior empirical support for improving *both* ART adherence and reducing substance use; and (2) feasibility of delivery by non-specialist health workers. These components included problem-solving strategies, BA, mindfulness training, and relapse prevention strategies. Each had also previously been delivered in isolation, in briefer interventions, and/or integrated with other skills (Andersen *et al*., 2018; McIntyre *et al*., 2018; Myers *et al*., 2019; Safren *et al*., 2021). These were then adapted for the setting based on key stakeholder input (Magidson *et al*., 2019, 2020*a*, 2020*b*, 2020*c*; Belus *et al*., 2020). The intervention manual was structured as a two-sided flipchart, providing the interventionist with reminders on key content and participants with visual aids (please contact authors for a copy).

*Khanya intervention components. Khanya* begins with problem-solving strategies to address ART adherence using a single-session problem-solving intervention [‘Life-Steps,’ previously adapted for the South African context, (Andersen *et al*., 2018)]. Specifically, participants (a) identify an adherence goal, (b) identify barriers to reaching the goal, and (c) create a plan and a back-up plan to support their goal (Safren *et al*., 1999). It was developed to help support ART adherence among men living with HIV in the United States and has frequently been integrated with other interventions, including cognitive behavioral therapy for depression (Safren *et al*., 2016, 2021). Life-Steps was further adapted for *Khanya* so that participants set a SU goal and to include a focus on how SU interfered with adherence (Magidson *et al*., 2020*b*), following prior work that has used problem solving to improve both ART adherence and SU in South Africa (Myers *et al*., 2019). BA increases awareness of individual's daily activities, behavioral patterns, and the links between mood, cravings and behavior, with a focus on increasing participation of value-driven, substance-free rewarding activities (Daughters *et al*., 2018). BA has previously been adapted and implemented in South Africa using task sharing (Andersen *et al*., 2018). Mindfulness training aims to increase awareness of the present moment with the aim of cultivating nonjudgmental awareness of negative affective states and reducing emotional avoidance (Miller, 1999). In *Khanya*, this includes both formal and informal mindfulness exercises that had been adapted to the South African context to promote feasibility and acceptability (McIntyre *et al*., 2018). Finally, relapse prevention strategies focus on the identification and navigation of high-risk situations (e.g. friends encouraging SU) (Marlatt and Gordon, 1985) and specific skills for the continued practice of the intervention components. The interventionist provided optional home practice activities to complete between sessions and kept materials at the study office if participants were concerned about disclosure in the home. Participants were able to participate in optional booster sessions (up to six), tailored as needed to each participant, after their post-treatment assessment. Table 1 and other recent papers (Belus *et al*., 2020; Magidson *et al*., 2020*b*) provide more detail on *Khanya* and interventionist supervision.

**Table 1.** Khanya intervention manual key components by session

Session number	Main focus	Content
1	Life-Steps	Check in on adherence Motivational strategies: Why is ARV adherence important? Goals for adherence Barriers to adherence Plan for overcoming barriers Review and home practice
2	Behavioral activation	Check in and home practice review Setting healthy goals Overview of behavioral activation and rationale Behavior monitoring Mindfulness exercise Review and home practice
3	Behavioral activation	Check in and home practice review Review behavioral activation rationale and monitoring Identifying activities Life areas and values exercise Activity scheduling Mindfulness exercise Review and home practice
4	Mindfulness training	Check in and home practice review Introduction to mindfulness in daily life Mindfulness for urges and cravings Mindfulness exercise Review and home practice
5	Relapse prevention strategies	Check in and home practice review Review mindfulness skills Identify high-risk situations Mindfulness exercise Review and home practice
6	Relapse prevention strategies	Check in and home practice review Maintaining goals/relapse prevention Review skills Plan for continued practice Mindfulness exercise Final wrap up
Optional booster(s)	Tailored as needed	Check in and home practice review Review skills, including barriers and facilitators Plan for continued practice

### Procedures

#### Qualitative interviews

Semi-structured, individual interviews with participants who had received *Khanya* were conducted by trained research assistants who had not been involved in delivering the intervention and who had received both prior and study-specific interviewing training. The interview guide (supplementary materials) explored perceptions of the overall implementation outcomes of the intervention, defined by Proctor's model (Proctor *et al*., 2011) and of specific intervention components. Acceptability was defined as satisfaction with various aspects of the intervention. Feasibility was defined as practicality for regular use, and appropriateness was defined as the usefulness and relevance for the setting. Of the thirty *Khanya* participants, two were deceased and three could not be reached; we conducted audio-recorded interviews with the remaining 25. The sound quality was too poor to be transcribed for two interviews, resulting in a final sample of *n* *=* 23. Interviews were translated from isiXhosa (the dominant local language) to English before transcription.

We also conducted semi-structured individual interviews with providers working at the clinic or co-located SU treatment program (*n* = 9), including nurses, substance use counselors, adherence counselors, supervisors, and administrators. The interview guide (supplementary materials) focused on perceptions of *Khanya* and its suitability for their health system and patients.

#### Quantitative surveys

Participants were asked to rank order the treatment components that had been most helpful to them. The purpose of asking patients to rank components relative to one another instead of rate each component independently was to avoid potential ceiling effects, which are known to occur with patient satisfaction rating scales in health care (Salman *et al*., 2020). Participants who did not receive all intervention components due to treatment non-completion were excluded; all four components were ranked by *n* = 20 at post-treatment and *n* = 22 at follow-up.

#### Qualitative analysis

Initial coding meetings included deductively identifying codes from the interview guide, while also inductively identifying additional codes. This allowed for coding to remain focused on perceptions of the intervention and its intervention components while ensuring any related, organically arising content was not missed (Fereday and Muir-Cochrane, 2006; Roberts *et al*., 2019). Qualitative interviews were then double coded by two members of the study team, working with a third team member who acted as an arbiter as needed. Coders obtained a Kappa score of 0.86 across transcripts, indicating good inter-coder reliability (O'Connor and Joffe, 2020). NVivo v.12 (QSR International Pty Ltd, 2020) was used for data management. Using a thematic analysis approach (Braun and Clarke, 2006), relationships between codes were then examined and cross-cutting themes were identified in discussion among the coding team, both about the overall intervention (patient interviews and provider interviews) and for specific intervention components (patient interviews only).

#### Quantitative analysis

For quantitative surveys, descriptive statistics were calculated (count and percentage) to identify rankings, from first to last, for intervention components at both timepoints.

#### Triangulation of qualitative and quantitative data sources

After separately examining qualitative and quantitative findings, data sources were integrated to examine the convergence and divergence of patient perspectives using an integrated visual joint display table. Among mixed methods designs, triangulation is best used when qualitative and quantitative data are collected simultaneously and can each offer information on the same question. In this case, both quantitative and qualitative data were able to provide information on which intervention components patients found most salient. Triangulation of findings across methods therefore helps bolster confidence in findings when there is convergence between methods and identify areas where clarity is lacking when there is a divergence between methods (O'Cathain *et al*., 2010). Convergence and divergence were examined at the group level, due to variable numbers of patients participating in qualitative and quantitative data collection (Fetters *et al*., 2013). Results were compiled using an integrated visual joint display table in which qualitative and quantitative results are presented side by side to illustrate convergence and divergence (McCrudden *et al*., 2021). Lastly, though qualitative data were not collected with the intention of explaining quantitative findings, qualitative data were able to provide preliminary complementary information on *why* intervention components were salient to patients and were examined for this purpose.

## Results

### Demographics

Patient participants were 48% female, 96% Black African, and had an average age of 39.2 (s.d. = 10.6). Provider participants were 56% female, 89% Black African, and had an average age of 40.8 (s.d. = 6.6).

### Qualitative patient perspectives on the overall *Khanya* intervention

Almost all patients described the overall *Khanya* intervention package as being acceptable, feasible, and appropriate for their needs. Participants spoke of *Khanya* as transformational and having a large impact on their quality of life. One patient shared:

‘*Now, I have a picture of a house that is tumbling down or it has been demolished. That's how my life was. And then when I came to Khanya it's like my, my, my, this house been built from foundation up.*’

- *Male, mid-30s*

Delivery of *Khanya* by a peer reportedly enhanced its acceptability and appropriateness. Some participants described that knowing that the interventionist shared lived experiences with them both made it easier to share their experiences with her and helped them feel heard when doing so. One participant stated:

*‘I was able to share whatever I wanted to share with her, you know, my problems… if I'm sharing something about myself, she would tell me that, you know, I've also experienced, um, something like that.’*

- *Female, mid-20s*

Although overall participants described *Khanya* to be feasible to participate in, some challenges were noted. For instance, in some situations, participants struggled with finding private space or time to complete the home practice components of *Khanya*. In some cases, this was because participants had not disclosed their HIV status to those they lived with. One participant who had not disclosed her HIV status to her partner said:

*‘At first I could not do things because my boyfriend was around… I did not do anything [home practice] at that time because my boyfriend was always you know around me’*

- *Female, early 30s*

Some participants described difficulty with attending weekly *Khanya* sessions due to job schedules. One participant shared:

*‘Since I'm self-employed, it did kind of get in the way of having to come to the sessions because I had to get jobs that I need to quickly take… because I need to get money.’*

- *Male, early 40s*

Participants reported that *Khanya* largely met their needs. In addition, there were several topics that participants suggested it would be helpful to add in the future. These included additional information on both SU and HIV and support around disclosure of HIV status to close relations, which was not a focus of the intervention. One participant said:

*‘I want to tell my partner but I'm not sure when to tell my-my partner about my status. I do not know when is the right time, or how-how to tell my partner. I'm not sure if this, uhm, in connection with the therapy. I don't know, but this is something I would like to get more advice on.’*

- *Female, early 40s*

### Qualitative provider perspectives on the overall *Khanya* intervention

Although none of the providers interviewed were directly involved with delivering the *Khanya* intervention, several noted that *Khanya* met the needs of their patients and noted improvements in HIV and SU outcomes among *Khanya* participants.

*‘Yes, [the Khanya participants] have improved on the [ARV] default rate, they are able to adhere well. Some others they were helped so much that they are no longer using any substances now…’’*

– *Female, Clinical Nurse Practitioner in HIV care, early 50s*

Providers noted the importance of the intervention being delivered by a peer. Not only does a peer bring vital shared lived experiences, but providers also reported that their capacity to deliver an intervention like *Khanya* was limited within the context of existing responsibilities. One provider said:

*‘It's just the business of the clinic, you know, unless it can, like for instance, there could be allocated time for this and maybe make a plan on what, how much time or what will I be doing at a certain time because the clinic is busy and we are already short staffed.’*

- *Female, Enrolled Nurse in SU care, late 30s*

### Triangulation of qualitative and quantitative patient perceptions of specific *Khanya* intervention components

Table 2 presents an integrated visual joint display table with the quantitative rankings of *Khanya* intervention components alongside representative patient quotes on each intervention component and an assessment of convergence and divergence across methods.

**Table 2.** Integrated visual joint display table displaying quantitative and qualitative findings and convergence and divergence between qualitative and quantitative methods

Intervention component	Patient quantitative ranking at PT (*n* = 20)	Patient quantitative ranking at 6MFU (*n* = 22)	Representative quote from patient (*n* = 23)	Convergence and divergence
Life-Steps	1*(*n* = 6, 30% of participants ranked it as most helpful)	2 (*n* = 6, 27.3%)	*‘So, this ABC are also included in in the Life-Steps, whereby A stands for the plan and then B stands for the barriers, um, and then when you are experiencing these barriers, what is it that you can do to overcome them? And then, just to make an example, if it might rain, you know, what can I do to make sure that I-I-I take my medication at the clinic. So, rain in itself is a barrier for me. So, we try to find out, um, different ways of overcoming that barrier and, or maybe a plan that you can- you can- you can have, you know, in order for you to make sure that you do get your medication at the clinic and for me was to take a taxi oh which it's it really worked for me.’* *– Male, late 30s*	Participants ranked relatively highly at both time points and many described use of skill spontaneously in qualitative interviews
Mindfulness training	1* (*n* = 6, 30% of participants ranked it as most helpful)	1 (*n* = 9, 41.0%)	*‘I don't really know how to explain it but you use it when you are very angry or maybe someone has done something to you that you don't like, you use it… It's about you know breathing in and also breathing out and also you know keeping attention to the things that we do like uh when you are cooking you need to pay attention to what you are doing. And also tasting your food or maybe you are drinking tea; I need to be aware of what I am doing and also sipping the tea and also tasting the tea.’* *- Female, early 40s*	Participants ranked relatively highly at first time point, more highly at second time point, and many described use of skill spontaneously in qualitative interviews
Behavioral activation	1* (*n* = 6, 30% of participants ranked it as most helpful)	3 (*n* = 4, 18.2%)	*‘So usually when I crave, I would go out and drink alcohol, but when I would be at home and find out that I'm craving I will not go out and drink alcohol. I would be busy, I would make myself busy in the house and not drink alcohol.’* *Male, mid-50s*	Participants ranked relatively highly at first time point, lower at second time point, and few described use of skill spontaneously in qualitative interviews
Relapse prevention skills	4 (*n* = 2, 10% of participants ranked it as most helpful)	4 (*n* = 3, 13.6%)	*‘Usually the people that I would spend a drink with I'd spend my time drinking alcohol with and then I would find myself ending up drinking with them and I can't say no, I can't say no so I'll end up drinking. But then if I'm not with them or they're not there then, I'm fine I don't even think about drinking I don't even drink I just don't drink at all… I was very much able to do that to identify such situations that would lead me back drinking the way like heavily.’* *Male, early 50s*	Few participants ranked highly at either time point or described use of skill spontaneously in qualitative interviews

PT, post-treatment assessment; 6MFU, six-month follow up assessment; *, tied with other components.

#### Mindfulness

Quantitatively, mindfulness training was tied with Life-Steps and BA for first (most useful) at three months (*n* = 6, 30.0%) and ranked first (*n* = 9, 41.0%) at the six-month follow up. Qualitatively, mindfulness training was also described in many interviews as being the most feasible, acceptable, and appropriate intervention component. For mindfulness training, feasibility and appropriateness were linked. Patients described the fact that they could integrate mindfulness training into their daily activities of living as being central to their perceptions of its appropriateness. Some patients also described that integrating mindfulness training directly into daily activities increased their enjoyment of the daily activities:

*‘It has taught me to-to be focused and to be mindful of what I'm doing. For instance, if I'm cooking I need to, you know, focus on what I am doing at that time. If I'm stirring the pot and I would, you know, I would stir it properly and be aware that, okay I'm stirring my pot, this is what I'm doing at this moment… I was able to do these skills and to, you know, practice them and also finish what I was doing, you understand.’*

- *Female, early 40s*

Patients also spoke of how it was easy to continue with mindfulness skills after the end of the intervention, because it fit well within their existing activities, reflecting its top ranking at the six-month follow up:

*‘I try by all means even though it might not be the same as what she had taught me but I try by all means to use that one [mindfulness] also.’*

- *Female, mid-40s*

#### Life-Steps

Quantitatively, Life-Steps was also ranked as a particularly helpful *Khanya* component, chosen first by six patients at both three-month (30.0%) and six-month (27.3%) follow ups. Qualitatively, Life-Steps was identified as a very acceptable, feasible, and appropriate aspect of *Khanya* in many of the interviews. Patients described that identifying their personal barriers to adherence was useful because it allowed for the development of individualized, instead of general, plans to support adherence. Patients also described that they appreciated the fact that Life-Steps allowed them to develop multiple plans to manage an adherence challenge. The development of plans and back up plans increased their confidence in their ability to manage a situation even in the face of an initial plan not working. Patients said:

*‘I will figure something else, you see sister [Life-Steps] was…just to show that actually you are not just giving up, you keep on trying and have a plan, another plan, another plan, another plan over and over.’*

*– Male, early 30s*

#### Behavioral activation and relapse prevention

All intervention components were ranked as most useful by at least one participant. BA and relapse prevention strategies were in general ranked less highly by participants and appeared less often in the qualitative interviews. However, some participants spoke of their value. Of BA, one participant said:

*‘I also asked myself you know, certain questions like, what is it that I want from life? What is my purpose for life? You know what are my values? What is it that I want to have in this life, things like that.’*

*- Male, mid-30s*

Of relapse prevention strategies, another participant said:

*“They were drinking alcohol and then they would tell me, “[name] come, you should come and join us”. But since I knew .., I had gone there to do the job and to work so I was able to tell myself, “No. You're not supposed to drink you are here to work and you must finish your job and then go back home.”*

- *Male, late 30s*

As indicated in Table 2, we found overall strong convergence of findings across methods. Mindfulness training and Life-Steps were quantitatively ranked as top intervention components and rich qualitative descriptions were provided by many patient participants about the acceptability, feasibility, and appropriateness of these components. Patients initially ranked BA the same as Life-Steps and mindfulness training yet provided fewer rich descriptions of their experience with BA, demonstrating some differences between quantitative and qualitative methods for this component. Relapse prevention strategies were ranked lowest at both time points and few patient participants discussed it qualitatively, again revealing strong convergence across methods on this topic.

## Discussion

This study identified several key findings related to our primary aims of exploring perspectives on the implementation outcomes of *Khanya* and its specific components. Findings demonstrated that patients and providers perceived *Khanya* as overall acceptable, appropriate, and feasible. But some reported feasibility challenges, including with home practice. Second, mixed methods analysis demonstrated a range in how intervention components were perceived, with mindfulness training and Life-Steps identified as particularly acceptable, feasible, and appropriate by participants and relapse prevention strategies as potentially less so. Results on BA were less consistent across methods. Findings underscore the value of more granular examination of implementation outcomes and of examining outcomes of specific intervention components in addition to entire packages.

Although patient perspectives were overall positive regarding *Khanya*, a detailed analysis of implementation outcomes revealed variety in the patient perspectives of specific components and some difficulties with feasibility. Though brief quantitative measures, for instance the Applied Mental Health Research group (AMHR) measure used in our clinical trial (Magidson *et al*., 2021), can help increase consistency and rigor of implementation outcomes reporting, there may also be ceiling effects within implementation outcome measures, in which ratings skew high. In general, better psychometric data is needed on quantitative implementation outcomes measures (Mettert *et al*., 2020). This also highlights the importance of qualitative data within implementation science (Hamilton and Finley, 2019). Of note, the intervention's delivery by a peer contributed to positive patient and provider impressions. Providers reported they did not have the time to take on an intervention like *Khanya*. This suggests that not only are peer interventionists potentially acceptable and appropriate from a patient perspective but that they may offer a way of expanding the behavioral health workforce in LMICs. Leveraging the potential for peer interventionists to extend access to SU services will require the resolution of issues around the roles, training and funding of lay health workers within health systems (Masis *et al*., 2021).

Some intervention components were perceived more positively and as more salient than others. The positive patient response to Life-Steps is consistent with the broader HIV literature that has established the implementation outcomes of Life-Steps among several samples of PLWH, including samples using substances (Safren *et al*., 1999; Magidson *et al*., 2014; Mimiaga *et al*., 2019). In contrast, mindfulness training was the most novel psychotherapy component for the setting. Adaptation of mindfulness training to sub-Saharan Africa has been relatively limited to date and has focused on mental health outcomes (i.e. depression, anxiety, and stress) instead of SU or HIV outcomes (McIntyre *et al*., 2018; Geda *et al*., 2021; Musa *et al*., 2021). Mindfulness training is infrequently included in task-shared psychological interventions in general (Wagenaar *et al*., 2020). The positive patient response to mindfulness training was seemingly driven by the feasibility of informally using these strategies within daily life and in settings where space and privacy may be a concern, a finding that echoes results of prior work in South Africa (McIntyre *et al*., 2018). In sum, the positive participant response to mindfulness training in this sample holds promise for the further exploration and testing of mindfulness-based approaches as part of task-shared behavioral interventions. However, there were also participants who ranked mindfulness training lowest. Better understanding the factors that contribute to the diversity of responses to intervention components may help guide the development of personalized task-shared behavioral health interventions (Ng and Weisz, 2016) and is an important direction for future research.

Though BA and relapse prevention strategies may have been less salient for *Khanya* participants, this does not necessarily mean that participants disliked these components. The use of forced-choice rankings, though intended to reduce ceiling effects, may have capped the results for these components. Notably, BA appeared to have stronger results quantitatively than qualitatively. It may be possible that patients had a harder time describing this intervention component. Using BA visuals during the interview, as was done during the intervention, may have yielded different qualitative results. Further, we included participants based on SU and not depression criteria, which may have somewhat undermined the salience of BA. There is other evidence in support of the implementation outcomes of BA in LMICs; a recent systematic review reported good acceptability, appropriateness, and feasibility of cognitive behavioral therapy, including BA, across ten countries (Verhey *et al*., 2020). In contrast, there is somewhat less support for the implementation outcomes of relapse prevention strategies in LMICs. A recent narrative review of relapse prevention in LMICs including studies across eight countries identified the need to increase the acceptability of relapse prevention to female patients, to focus more on encouraging help-seeking, and to better tailor descriptions of SU patterns to local settings (Heijdra Suasnabar and Hipple Walters, 2020). Within *Khanya*, relapse prevention strategies are only introduced in the final two sessions, so it is also possible there was insufficient time to practice this skill within the intervention. It is also possible that in a sample of participants with more severe SU, relapse prevention strategies would have had more salience.

There are several limitations to this study that are important to note. Among these is the fact that we have quantitative data from two timepoints, and qualitative data from only one, meaning we have a limited ability to draw conclusions on why patient rankings of specific components changed over time. We also had a limited sample size of intervention participants. However, our sample does represent slightly more than three quarters of those receiving *Khanya* and we use multiple methods convergently to bolster our findings. Additionally, as discussed above, the use of forced-choice rankings may have influenced findings. However, again we use qualitative data to reinforce quantitative findings. Lastly, only one peer interventionist delivered *Khanya*, which means we were not able to explore the acceptability, feasibility, and appropriateness of *Khanya* among peer providers. Future work will examine delivery by multiple peers, explore any challenges that might arise with broader peer delivery of *Khanya,* and explore strategies for articulating peer roles and supporting peers in South Africa.

## Conclusions

Our findings highlight the use of examining perspectives on the specific intervention components of behavioral interventions as well as on overall intervention packages. Perspectives on the components within *Khanya* vary, which should be further explored in future research and could inform future adaptations and delivery of *Khanya* and other similar packages. Findings suggest that peer models and other skills not typically included in task-sharing interventions, such as mindfulness training, may be well-perceived by patients and providers and indicate possible avenues for intervention innovation.

## Supplementary material

For supplementary material accompanying this paper visit https://doi.org/10.1017/gmh.2022.47. click here to view supplementary material

## References

[ref1] Andersen LS, Magidson JF, O'Cleirigh C, Remmert JE, Kagee A, Leaver M, Stein DJ, Safren SA and Joska J (2018) A pilot study of a nurse-delivered cognitive behavioral therapy intervention (Ziphamandla) for adherence and depression in HIV in South Africa. Journal of Health Psychology 23, 776–787. 27121977 10.1177/1359105316643375 PMC5081274

[ref2] Belus JM, Rose AL, Andersen LS, Ciya N, Joska JA, Myers B, Safren SA and Magidson JF (2020) Adapting a behavioral intervention for alcohol use and HIV medication adherence for lay counselor delivery in Cape Town, South Africa: a case series. Cognitive and Behavioral Practice 29, 454–467. 10.1016/j.cbpra.2020.10.003 36171964 PMC9512118

[ref3] Boustani MM, Frazier SL, Chu W, Lesperance N, Becker KD, Helseth SA, Hedemann ER, Ogle RR and Chorpita BF (2020) Common elements of childhood universal mental health programming. Administration and Policy in Mental Health and Mental Health Services Research 47, 475–486. 32080783 10.1007/s10488-020-01023-4

[ref4] Braun V, and Clarke V (2006) Using thematic analysis in psychology. Qualitative Research in Psychology 3, 77–101.

[ref5] Bruckner TA, Scheffler RM, Shen G, Yoon J, Chisholm D, Morris J, Fulton BD, Dal Poz MR and Saxena S (2011) The mental health workforce gap in low- and middle-income countries: a needs-based approach. Bulletin of the World Health Organization 89, 184–194. 10.2471/BLT.10.082784 21379414 PMC3044251

[ref6] Daughters SB, Magidson JF, Anand D, Seitz-Brown CJ, Chen Y and Baker S (2018) The effect of a behavioral activation treatment for substance use on post-treatment abstinence: a randomized controlled trial. Addiction Abingdon Engl. 113, 535–544. 10.1111/add.14049 PMC5807178 28963853

[ref7] Domhardt M, Geßlein H, von Rezori RE and Baumeister H (2019) Internet- and mobile-based interventions for anxiety disorders: a meta-analytic review of intervention components. Depression and Anxiety 36, 213–224. 30450811 10.1002/da.22860

[ref8] Eaton AD, Carusone SC, Craig SL, Telegdi E, McCullagh JW, McClure D, Wilson W, Zuniga L, Berney K, Ginocchio GF, Wells GA, Montess M, Busch A, Boyce N, Strike C and Stewart A (2019) The ART of conversation: feasibility and acceptability of a pilot peer intervention to help transition complex HIV-positive people from hospital to community. BMJ Open 9, e026674. 10.1136/bmjopen-2018-026674 PMC6475144 30928956

[ref9] Fereday J and Muir-Cochrane E (2006) Demonstrating rigor using thematic analysis: a hybrid approach of inductive and deductive coding and theme development. International Journal of Qualitative Methods 5, 80–92.

[ref10] Fetters MD, Curry LA and Creswell JW (2013) Achieving integration in mixed methods designs – principles and practices. Health Services Research 48, 2134–2156. 24279835 10.1111/1475-6773.12117 PMC4097839

[ref11] Geda YE, Krell-Roesch J, Fisseha Y, Tefera A, Beyero T, Rosenbaum D, Szabo TG, Araya M and Hayes SC (2021) Acceptance and commitment therapy in a low-income country in sub-Saharan Africa: a call for further research. Frontiers in Public Health 9, 732800. 34631649 10.3389/fpubh.2021.732800 PMC8494766

[ref12] Hamilton AB and Finley EP (2019) Qualitative methods in implementation research: an introduction. Psychiatry Research 280, 112516. 31437661 10.1016/j.psychres.2019.112516 PMC7023962

[ref13] Heijdra Suasnabar JM and Hipple Walters B (2020) Community-based psychosocial substance use disorder interventions in low-and-middle-income countries: a narrative literature review. International Journal of Mental Health Systems 14, 74. 33062049 10.1186/s13033-020-00405-3 PMC7542947

[ref14] Hoeft TJ, Fortney JC, Patel V and Unützer J (2018) Task-sharing approaches to improve mental health care in rural and other low-resource settings: a systematic review. The Journal of Rural Health 34, 48–62. 28084667 10.1111/jrh.12229 PMC5509535

[ref15] Kader R, Seedat S, Govender R, Koch JR and Parry CD (2014) Hazardous and harmful use of alcohol and/or other drugs and health status among South African patients attending HIV clinics. AIDS and Behavior 18, 525–534. 23921585 10.1007/s10461-013-0587-9

[ref16] Kekwaletswe CT and Morojele NK (2014) Alcohol use, antiretroviral therapy adherence, and preferences regarding an alcohol-focused adherence intervention in patients with human immunodeficiency virus. Patient Preference and Adherence 8, 401–413. 24729688 10.2147/PPA.S55547 PMC3976236

[ref17] Kim H, Tanser F, Tomita A, Vandormael A and Cuadros DF (2021) Beyond HIV prevalence: identifying people living with HIV within underserved areas in South Africa. BMJ Global Health 6, e004089. 10.1136/bmjgh-2020-004089 PMC8061852 33883186

[ref18] Magidson JF, Seitz-Brown CJ, Safren SA and Daughters SB (2014) Implementing behavioral activation and life-steps for depression and HIV medication adherence in a community health center. Cognitive and Behavioral Practice 21, 386–403. 25419102 10.1016/j.cbpra.2013.10.002 PMC4238929

[ref19] Magidson JF, Joska JA, Regenauer KS, Satinsky E, Andersen LS, Seitz-Brown CJ, Borba CP, Safren SA and Myers B (2019) “Someone who is in this thing that I am suffering from”: the role of peers and other facilitators for task sharing substance use treatment in South African HIV care. The International Journal on Drug Policy 70, 61–69. 31082664 10.1016/j.drugpo.2018.11.004 PMC6679990

[ref20] Magidson JF, Andersen LS, Satinsky EN, Myers B, Kagee A, Anvari M and Joska JA (2020 *a*) “Too much boredom isn't a good thing”: adapting behavioral activation for substance use in a resource-limited South African HIV care setting. Psychotherapy 57, 107–118. 31670529 10.1037/pst0000257 PMC7069775

[ref21] Magidson JF, Joska JA, Myers B, Belus JM, Regenauer KS, Andersen LS, Majokweni S, O'Cleirigh C and Safren SA (2020 *b*) Project Khanya: a randomized, hybrid effectiveness-implementation trial of a peer-delivered behavioral intervention for ART adherence and substance use in Cape Town, South Africa. Implementation Science Communications 1, 23. 32607502 10.1186/s43058-020-00004-w PMC7326344

[ref22] Magidson JF, Satinsky EN, Luberto CM, Myers B, Funes CJ, Vanderkruik R and Andersen LS (2020 *c*) “Cooling of the mind”: assessing the relevance of mindfulness training among people living with HIV using alcohol and other substances in South Africa. Social Science & Medicine (1982) 1982(266), 113424. 10.1016/j.socscimed.2020.113424. PMC8183602 33065498

[ref23] Magidson JF, Joska JA, Belus JM, Andersen LS, Regenauer KS, Rose AL, Myers B, Majokweni S, O'Cleirigh C and Safren SA (2021) Project Khanya: results from a pilot randomized type 1 hybrid effectiveness-implementation trial of a peer-delivered behavioural intervention for ART adherence and substance use in HIV care in South Africa. Journal of the International AIDS Society 24(suppl. 2), e25720–e25720. 10.1002/jia2.25720. 34164935 PMC8222840

[ref24] Marlatt GA and Gordon JR (1985) Relapse Prevention: A Self-Control Strategy for the Maintenance of Behavior Change. New York: Guilford, pp. 85–101.

[ref25] Masis L, Gichaga A, Zerayacob T, Lu C and Perry HB (2021) Community health workers at the dawn of a new era: 4. Programme financing. Health Research Policy and Systems 19, 107. 34641893 10.1186/s12961-021-00751-9 PMC8506106

[ref26] McCrudden MT, Marchand G and Schutz PA (2021) Joint displays for mixed methods research in psychology. Methods in Psychology 5, 100067.

[ref27] McIntyre T-L, Elkonin D, de Kooker M and Magidson JF (2018) The application of mindfulness for individuals living with HIV in South Africa: a hybrid effectiveness-implementation pilot study. Mindfulness 9, 871–883. 30079121 PMC6070157

[ref28] Mettert K, Lewis C, Dorsey C, Halko H and Weiner B (2020) Measuring implementation outcomes: an updated systematic review of measures’ psychometric properties. Implementation Research and Practice 1, 2633489520936644. 10.1177/2633489520936644. PMC9924262 37089128

[ref29] Miller WR (1999) Integrating Spirituality Into Treatment: Resources for Practitioners. Washington, DC: American Psychological Association.

[ref30] Mimiaga MJ, Bogart LM, Thurston IB, Santostefano CM, Closson EF, Skeer MR, Biello KB and Safren SA (2019) Positive strategies to enhance problem-solving skills (STEPS): a pilot randomized, controlled trial of a multicomponent, technology-enhanced, customizable antiretroviral adherence intervention for HIV-infected adolescents and young adults. AIDS Patient Care and STDs 33, 21–24. 10.1089/apc.2018.0138. 30601059 PMC6338456

[ref31] Mira A, Soler C, Alda M, Baños R, Castilla D, Castro A, García-Campayo J, García-Palacios A, Gili M, Hurtado M, Mayoral F, Montero-Marín J and Botella C (2019) Exploring the relationship between the acceptability of an internet-based intervention for depression in primary care and clinical outcomes: secondary analysis of a randomized controlled trial. Frontiers in Psychiatry 10, 325–325. 10.3389/fpsyt.2019.00325. 31133899 PMC6523778

[ref32] Musa ZA, Soh KL, Mukhtar F, Soh KY, Oladele TO and Soh KG (2021) Effectiveness of mindfulness-based cognitive therapy among depressed individuals with disabilities in Nigeria: a randomized controlled trial. Psychiatry Research 296, 113680. 10.1016/j.psychres.2020.113680. 33421840

[ref33] Myers B, Joska JA, Lund C, Levitt NS, Butler CC, Naledi T, Milligan P, Stein DJ and Sorsdahl K (2018) Patient preferences for the integration of mental health counseling and chronic disease care in South Africa. Patient Preference and Adherence 12, 1797–1803. 10.2147/PPA.S176356. 30271123 PMC6154740

[ref34] Myers B, Petersen-Williams P, van der Westhuizen C, Lund C, Lombard C, Joska JA, Levitt NS, Butler C, Naledi T, Milligan P and Stein D (2019) Community health worker-delivered counselling for common mental disorders among chronic disease patients in South Africa: a feasibility study. BMJ Open 9, e024277. 10.1136/bmjopen-2018-024277 PMC6340481 30647043

[ref35] Necho M, Belete A and Getachew Y (2020) The prevalence and factors associated with alcohol use disorder among people living with HIV/AIDS in Africa: a systematic review and meta-analysis. Substance Abuse Treatment, Prevention, and Policy 15, 63. 10.1186/s13011-020-00301-6. 32831129 PMC7444054

[ref36] Ng MY and Weisz JR (2016) Building a science of personalized intervention for youth mental health. Journal of Child Psychology and Psychiatry, and Allied Disciplines 57, 216–236. 10.1111/jcpp.12470. 26467325 PMC4760855

[ref37] O'Cathain A, Murphy E and Nicholl J (2010) Three techniques for integrating data in mixed methods studies. BMJ 341, c4587. 10.1136/bmj.c4587. 20851841

[ref38] O'Connor C and Joffe H (2020) Intercoder reliability in qualitative research: debates and practical guidelines. International Journal of Qualitative Methods 19, 1609406919899220. 10.1177/1609406919899220.

[ref39] Proctor E, Silmere H, Raghavan R, Hovmand P, Aarons G, Bunger A, Griffey R and Hensley M (2011) Outcomes for implementation research: conceptual distinctions, measurement challenges, and research agenda. Administration and Policy in Mental Health 38, 65–76. 20957426 10.1007/s10488-010-0319-7 PMC3068522

[ref40] Proctor EK, Powell BJ and McMillen JC (2013) Implementation strategies: recommendations for specifying and reporting. Implementation Science 8, 139. 24289295 10.1186/1748-5908-8-139 PMC3882890

[ref41] QSR International Pty Ltd (2020) NVivo (released March 2020).

[ref42] Roberts K, Dowell A and Nie J-B (2019) Attempting rigour and replicability in thematic analysis of qualitative research data; a case study of codebook development. BMC Medical Research Methodology 19, 66. 30922220 10.1186/s12874-019-0707-y PMC6437927

[ref43] Safren SA, Otto MW and Worth JL (1999) Life-steps: applying cognitive-behavioral therapy to patient adherence in HIV medication treatment. Cognitive and Behavioral Practice 6, 332–341.

[ref44] Safren SA, Bedoya CA, O'Cleirigh C, Biello KB, Pinkston MM, Stein MD, Traeger L, Kojic E, Robbins GK, Lerner JA, Herman DS, Mimiaga MJ and Mayer KH (2016) Cognitive behavioural therapy for adherence and depression in patients with HIV: a three-arm randomised controlled trial. The Lancet. Hiv 3, e529–e538. 27658881 10.1016/S2352-3018(16)30053-4 PMC5321546

[ref45] Safren SA, O'Cleirigh C, Andersen LS, Magidson JF, Lee JS, Bainter SA, Musinguzi N, Simoni J, Kagee A and Joska JA (2021) Treating depression and improving adherence in HIV care with task-shared cognitive behavioural therapy in Khayelitsha, South Africa: a randomized controlled trial. Journal of the International AIDS Society 24, e25823. 34708929 10.1002/jia2.25823 PMC8552453

[ref46] Salman AA, Kopp BJ, Thomas JE, Ring D and Fatehi A (2020) What are the priming and ceiling effects of one experience measure on another? Journal of Patient Experience 7, 1755–1759. 33457640 10.1177/2374373520951670 PMC7786675

[ref47] Satinsky EN, Kleinman MB, Tralka HM, Jack HE, Myers B and Magidson JF (2021) Peer-delivered services for substance use in low- and middle-income countries: a systematic review. The International Journal on Drug Policy 95, 103252. 33892281 10.1016/j.drugpo.2021.103252

[ref48] Shalaby RAH and Agyapong VIO (2020) Peer support in mental health: literature review. JMIR Mental Health 7, e15572. 32357127 10.2196/15572 PMC7312261

[ref49] Singla DR, Raviola G and Patel V (2018) Scaling up psychological treatments for common mental disorders: a call to action: LETTERS TO THE EDITOR. World Psychiatry 17, 226–227. 29856556 10.1002/wps.20532 PMC5980618

[ref50] Verhey IJ, Ryan GK, Scherer N and Magidson JF (2020) Implementation outcomes of cognitive behavioural therapy delivered by non-specialists for common mental disorders and substance-use disorders in low- and middle-income countries: a systematic review. International Journal of Mental Health Systems 14, 40. 32514304 10.1186/s13033-020-00372-9 PMC7260765

[ref51] Wagenaar BH, Hammett WH, Jackson C, Atkins DL, Belus JM and Kemp CG (2020) Implementation outcomes and strategies for depression interventions in low- and middle-income countries: a systematic review. Global Mental Health 7, e7. 10.1017/gmh.2020.1. 32346482 PMC7176918

