# Supplementary material for: Patient and provider perceptions of a peer-delivered intervention (‘Khanya’) to improve anti-retroviral adherence and substance use in South Africa: a mixed methods analysis
Source: Glob Ment Health (Camb). 2022 Aug 26;9:439–47. doi: 10.1017/gmh.2022.47 (PMC9807005; doi:10.1017/gmh.2022.47)
Supplement: Supplementary file 1 [file S2054425122000474sup001.docx]

**Qualitative Interview Guide: Patient Version**

Introduction: This interview will take approximately 45 minutes. Please let me know if you need a break at any time. The reason for this interview is to hear about your perceptions of the Khanya therapy that you received. We would like to hear your honest opinion of what you feel was helpful and what was not helpful in the intervention. We would like to improve upon this treatment as best we can, so please tell us any and all thoughts that you have.

I will be audio-recording this session.  Your name will not be on the recording. None of the study team at the clinic, including Nonceba, who provided you with the treatment, will have access to your recording, nor your treatment team at the clinic. After the interview, the recording will be typed up into a transcript.  If you happen to refer to a name during the session, the name will be removed from the transcript. What you share during this interview will in no way affect your treatment at the clinic. Your responses in this interview will hopefully help us develop better treatments for people with HIV who also have substance use concerns, so we would appreciate if you can be as honest as you can in your responses.

Any time you want to stop the interview or have me turn off the recording, you can tell me, and we will stop.

Do you have any questions before we start?

*Note: This document is meant to be a guide for the interviewer to structure the interview; however, the interviewer is encouraged to follow the participant’s responses and probe to gather as accurate a sense of their perspectives as possible. The primary topic areas will stay the same, but the specific questions we expect to change over time as we gather perspectives.*

*(Turn on the recording)*

Acceptability

1. Tell me briefly about your experience meeting with the interventionist, Nonceba, for the Khanya therapy. How would you describe the experience?

Probe: What was it like for you?

1. What skills did you find most useful (or applicable to you) in the treatment (*by skills, we mean topics of the therapy sessions with the interventionist, Nonceba – please feel free to describe in your own words*)?

NB: do not prompt the participant with skills from the treatment. We would like to see what skills resonated with them by assessing what skills they bring up on their own.

1. Which skills (*or topics from the Khanya program)* have you practiced (*or tried on your own)* outside of session? *(Ask for examples)*
2. Which parts of the Khanya program still help you today? *(Ask for examples)*
   1. Probe: Can you provide a recent example of a way that the Khanya therapy helped you in your life?
   2. Probe: Can you describe a recent example of a time that you used a skill from the Khanya program? *(Ask for examples across both adherence and substance use)*
3. How satisfied are you with your experience in the Khanya intervention (*i.e., by intervention, we are referring to the material you focused on with the interventionist, Nonceba)*? *Please explain why or why not you were satisfied.*

Therapeutic alliance:

1. Tell me about your relationship with the interventionist (Nonceba)
   1. Probe: *What are examples of how she was effective, or helpful, in your work together?*
   2. Probe: *What are examples of how she could improve?*

Appropriateness

1. How much did the treatment meet your needs? By that we mean, how much did the therapy focus on areas that were useful to you?

For Reference (provide participant with the handout of skills):

- - - Life Steps- develop a plan and back-up plan in order to overcome barriers to taking ARVs on time
    - Behavioural activation- the cycle of emotions, urges, and behaviours
    - Monitoring and scheduling substance-free activities
    - Identifying values- life areas that are important or valuable to you
    - Mindfulness- focusing on the present moment without judgment (includes mindfulness of everyday activities, urge surfing, SOBER breathing space)
    - Identifying high-risk situations to prevent using substances again or forgetting to take your ARVs regularly
    - Plan for continued practice of the skills learned after therapy ends

1. Tell me about areas the therapy focused on that were NOT useful to you? Please describe parts of the treatment that were not helpful to you.
   1. Probe: Why were they not helpful? What were those parts of the program like for you?
2. Is there any content related to substance use or adherence you would like to have added to the intervention?
3. Are any of the skills familiar to you because of something you were already doing?
   1. Clarification: *If unclear what is meant, say,* in other words, did the interventionist try to teach you something that you already knew?
   2. Probe: What words or phrases in the local language here would you use to describe what you learned in the Khanya intervention?
4. Are there any changes you would suggest making to the intervention to make it more consistent with your culture and language?
5. How have the people in your life -- friends, family, romantic partner, community members, played a role in your substance use (in a helpful OR harmful way)?
   1. Probe: *If not mentioned previously, probe about romantic partners specifically.*
6. How have the people in your life -- friends, family, romantic partner, community members, played a role in your HIV medication adherence (in a helpful OR harmful way)?
   1. Probe: *If not mentioned previously, probe about romantic partners specifically.*
7. How would you feel about participating in this type of intervention with a friend, family member, romantic partner, or community member?
   1. Probe: If you are comfortable, is there a specific person in your life that could support you in this way? What type of relationship do you have with this person?
   2. Probe: How could this person(s) be of greatest support to you?

Feasibility

1. Please describe how getting to the sessions were for you each week.
   1. Probe: What difficulties did you have in attending the treatment sessions? *Please provide an example.*
   2. Probe: What made it easier for you to attend the sessions? *Please provide an example.*
   3. Probe: How did you feel about coming to sessions once per week?
2. Please describe any challenges with home practice. By home practice, we mean practicing the skills you learned in the therapy outside of sessions (for instance, skills to support you in taking your medications, scheduling activities, mindfulness). What got in the way of practicing skills at home? [show participant reference sheet to skills]
   1. Probe: *Please provide an example of something that made it challenging to practice the skills outside of session.*
3. What made it easier to practice the therapy skills outside of session?
   1. Probe: *Please provide an example of something that made it easier to practice the skills outside of session.*
4. Please describe any challenges with using the Wisepill device. This is the device that you were asked to store your ARVs in during the study.
   1. Probe: *Please provide an example of something that made it challenging to use your Wisepill device.*
   2. Probe: *Can you tell me about a time that you did not use your Wisepill device on purpose?*
5. Please describe any challenges with using the Wisepill device in front of your family, friends, or others you live with.
   1. Probe: *Can you tell me about a time that you did not use your Wisepill device in front of family, friends, or others you live with?*
6. Please describe ways that the Wisepill device helped you to take your ARVs.
   1. Probe: *Please provide an example of a time when using the Wisepill device made it easy to take your medications.*
   2. Probe: *What about the Wisepill device helped you, if at all, to take your ARVs?*

Substance use questions:

1. How has your alcohol (or other drug use) changed, if at all, during the study?
   1. Probe: *What is an example of how it has changed?*
   2. Probe: *What has contributed to this change?*

*If amount or frequency of alcohol or other drug use has not changed…*

- 1. Probe: *What do you think would need to happen for your alcohol (or other drug use) to change?*
  2. Probe: *How have any problems related to alcohol (or other drug use) changed during the study?*

Adherence questions:

1. How has your ART adherence changed, if at all, during the study?
   1. Probe: *What is an example of how it has changed?*
   2. Probe: *What has contributed to this change?*

*If ART adherence has not changed…*

- 1. Probe: *What do you think would need to happen for your adherence to change?*
  2. Probe: *What still gets in the way of making this change?*

Wrap-Up

1. Please share any other feedback or thoughts you have on the treatment or that you would like to share.

*Thank you for your time and for sharing your insights. We hope to use this information to improve our program and we really value your input.*

**Qualitative Interview Guide: Provider Version**

This interview will take approximately 45 minutes. Please let me know if you need a break at any time. The reason for this interview is to better understand your perspective as a provider at Matrix and how we can better provide substance use treatment for patients living with HIV. We are interested to know how we can integrate ARV adherence support for people living with HIV into existing substance use treatment.

I will be audio-recording this session. Your name will not be on the recording. After the interview, the recording will be typed up into a transcript. If you happen to refer to a name during the session, the name will be removed from the transcript. What you share during this interview will in no way affect your role at Matrix. Your responses in this interview will hopefully help us develop better treatments for people living with HIV who also use substances, so we would appreciate if you can be as honest as you can in your responses.

Any time you want to stop the interview or have me turn off the recording, you can tell me, and we will stop.

Do you have any questions before we start?

*Note: This document is meant to be a guide for the interviewer to structure the interview; however, the interviewer is encouraged to follow the participant’s responses and probe to gather as accurate a sense of their perspectives as possible. The primary topic areas will stay the same, but the specific questions we expect to change over time as we gather perspectives.*

*(Turn on the recording)*

Introduction:

1. I’d like to start by telling you about the treatment we developed called “Khanya.” Are you aware of our study at Town 2?
   1. *Probe (if yes):* What have you heard about it? What are your thoughts on [reference what they know/have heard about Khanya]?
   2. *Probe (if yes):* Do you know any patients who were in Khanya? Tell me about what you noticed in patients who received the Khanya intervention.

Let me tell you a little bit more about Khanya. First, the name translates to ‘glow, direction, or light’ in isiXhosa. The treatment is 6 sessions and is designed to help patients living with HIV who are nonadherent to their ARVs AND who actively use alcohol or other substances, such as marijuana or tik. The treatment teaches specific behavioural skills to help patients improve their adherence to their ARVs and change their substance use.

Feasibility, Barriers/Facilitators of Uptake

1. Let me tell you more about the HIV treatment component, which is called Life Steps. Life Steps is a brief behavioural intervention where we discuss a patient’s adherence goals, the challenges or barriers that stand in their way of reaching those goals, and then we come up with plans that help them overcome the barriers so that they can adhere to their ARVs every day. We go through some of the most common barriers to taking ARVs such as transportation, forgetting, or alcohol use.
   1. What are your overall reactions to the idea of integrating Life Steps into Matrix?
2. What are some of the barriers that would make it harder to integrate Life Steps into Matrix?
   1. *Probe. What are some patient barriers?*
   2. *Probe. What are some provider barriers?*
   3. *Probe. What are some clinic-level barriers?*
3. What are some facilitators, or things that would make it easier, to integrate Life Steps into Matrix?
   1. *Probe. For patients?*
   2. *Probe. For providers?*
   3. *Probe. For the clinic?*
4. Please describe how we could support Matrix providers to deliver interventions for HIV treatment adherence as part of their role.
   1. *Probe.* *What would get in the way?*
   2. *Probe. What would make it easier?*

Feedback on Preliminary Results

1. We wanted to share some preliminary results from our Khanya study and get your feedback on them. Half of our sample received Khanya, a 6-session treatment. The other half were given a referral to Matrix and walked over. We found that patients who attended the Khanya intervention and patients who were referred to Matrix both improved their substance use. However, only the patients in Khanya improved their HIV medication adherence. What are your reactions to this finding?
   1. *Probe.* *What are your reactions to the substance use findings, that both groups, including those who received Matrix improved?*
   2. *Probe. What are your reactions to the adherence findings, that only the Khanya group, not the Matrix only group improved?*
2. We found that most patients who were referred to Matrix through the study attended the intake appointment, but did not attend any follow-up visits. Why do you think this is?
   1. Probe: *Tell me how this is similar or different from what you see in clinical practice at Matrix.*
   2. Probe: *Why do patients drop out of care after the intake appointment?*
3. What are strategies that you or your colleagues use to increase patient engagement and attendance at Matrix, especially when patients first start treatment?
   1. *Probe: What do you think patients need to stay engaged in care?*
4. Although the majority of patients only attended the intake session, we did see improvements in substance use among patients, even after only attending the intake and then completing assessments with our team. What is your reaction to this finding?
   1. *Probe: Why do you think these patients still improved, even though they did not complete the Matrix program?*
   2. *Probe:* *Is it ever the case that patients attend the intake assessment and that, in and of itself, is an intervention that gets them to change their behaviour? Tell me more about that.*

HIV adherence and referrals

1. How do Matrix providers currently support patients who are not adhering to their HIV treatment?
   1. *Probe: What is working well currently?*
   2. *Probe: What is not working well currently?*
   3. *Probe: What do providers need to be able to support patients who are not adhering to HIV treatment?*
2. Tell me what happens clinically when you work with a patient who has medical comorbidities, either HIV or something else, say TB or diabetes, and you think the patient has not been keeping up with their medical care.
   1. *Probe (if not already addressed): Do you ever make a referral for a patient to the Town II clinic for medical reasons?*
   2. *Probe: What information do you try and get from the patient to decide if they need a referral or need additional medical care?*
   3. *Probe: What information do you try and get elsewhere to decide if the patient needs additional medical care? For example, you might look in the patient’s chart or talk to the patient’s family members to get additional information.*
3. How would you describe the relationship between Matrix and Town II clinic in terms of coordinating care for shared patients?
   1. Probe. *What could be improved in order to provide patients with better care?*

Other factors that improve outcomes

1. Please describe efforts at Matrix to include family members or loved ones in care.
   1. *Probe: What works well in these sessions?*
   2. *Probe: What is challenging?*
   3. *Probe: How can family members/loved ones be part of support to improve HIV medication adherence for patients at Matrix?*

Wrap-Up

1. Please share any other feedback or thoughts you have on the topic we’ve been discussing.

Thank you for your time and for sharing your insights. We hope to use this information to improve our program and we really value your input.
